# Assessing the burden of bronchiolitis and lower respiratory tract infections in children ≤24 months of age in Italy, 2012–2019

**DOI:** 10.3389/fped.2023.1143735

**Published:** 2023-05-05

**Authors:** Elisa Barbieri, Sara Cavagnis, Antonio Scamarcia, Luigi Cantarutti, Lorenzo Bertizzolo, Mathieu Bangert, Salvatore Parisi, Anna Cantarutti, Eugenio Baraldi, Carlo Giaquinto, Vincenzo Baldo

**Affiliations:** ^1^ Division of Paediatric Infectious Diseases, Department for Women’s and Children’s Health, University of Padua, Padua, Italy; ^2^ Societa' Servizi Telematici - Pedianet, Padua, Italy; ^3^ Sanofi Vaccines, Lyon, France; ^4^ Sanofi Vaccines, Milan, Italy; ^5^ Unit of Biostatistics, Epidemiology and Public Health, Department of Statistics and Quantitative Methods, University of Milano-Bicocca, Milan, Italy; ^6^ National Centre for Healthcare Research and Pharmacoepidemiology, Department of Statistics and Quantitative Methods, University of Milano-Bicocca, Milan, Italy; ^7^ Neonatal Intensive Care Unit, Department of Women's and Children's Health, University of Padua, Padua, Italy; ^8^ Department of Cardiac Thoracic and Vascular Sciences and Public Health, University of Padua, Padua, Italy

**Keywords:** bronchiolitis, epidemiology, Italy, respiratory syncytial virus, children, lower respiratory tract infection (LRTI)

## Abstract

**Background:**

Bronchiolitis is the most common lower respiratory tract infection (LRTI) in children and is mainly caused by the Respiratory Syncytial Virus (RSV). Bronchiolitis presents seasonally and lasts about five months, usually between October to March, with peaks of hospitalizations between December and February, in the Northern Hemisphere. The burden of bronchiolitis and RSV in primary care is not well understood.

**Materials and methods:**

This retrospective analysis used data from Pedianet, a comprehensive paediatric primary care database of 161 family paediatricians in Italy. We evaluated the incidence rates (IR) of all-cause bronchiolitis (ICD9-CM codes 466.1, 466.11 or 466.19), all-cause LRTIs, RSV-bronchiolitis and RSV-LRTIs in children from 0 to 24 months of age, between January 2012 to December 2019. The role of prematurity (<37 weeks of gestational age) as a bronchiolitis risk factor was evaluated and expressed as odds ratio.

**Results:**

Of the 108,960 children included in the study cohort, 7,956 episodes of bronchiolitis and 37,827 episodes of LRTIs were recorded for an IR of 47 and 221 × 1,000 person-years, respectively. IRs did not vary significantly throughout the eight years of RSV seasons considered, showing a seasonality usually lasting five months, between October and March, while the peak of incidence was between December and February. Bronchiolitis and LRTI IRs were higher during the RSV season, between October and March, regardless of the month of birth, with bronchiolitis IR being higher in children aged ≤12 months. Only 2.3% of bronchiolitis and LRTI were coded as RSV-related. Prematurity and comorbidity increased the risk of bronchiolitis; however, 92% of cases happened in children born at term, and 97% happened in children with no comorbidities or otherwise healthy.

**Conclusions:**

Our results confirm that all children aged ≤24 months are at risk of bronchiolitis and LRTI during the RSV season, regardless of the month of birth, gestational age or underlying health conditions. The IRs of bronchiolitis and LRTI RSV-related are underestimated due to the poor outpatient epidemiological and virological surveillance. Strengthening the surveillance system at the paediatric outpatient level, as well as at the inpatient level, is needed to unveil the actual burden of RSV-bronchiolitis and RSV-LRTI, as well as to evaluate the effectiveness of new preventive strategies for anti-RSV.

## Background

Respiratory Syncytial Virus (RSV) is the leading cause of hospitalization in children aged ≤12 months due to lower respiratory tract infection (LRTI), such as pneumonia and bronchiolitis, and represents a significant public health challenge worldwide (1). Between 50% to 80% of children hospitalized for bronchiolitis aged 0–5 years are positive for RSV, while the positivity to RSV rise to 80% in children aged ≤12 months (2); furthermore, 40% of children hospitalized for pneumonia aged ≤12 months are positive to RSV (3).

Almost every child is infected by RSV at least once by the second year of life. Children with an RSV infection require a medical visit at the paediatric practice in more than 20% of cases; 6% of them need medical assistance at the emergency department, and almost 4% require hospitalization (1, 4, 5).

The yearly global cost of inpatient and outpatient RSV in children <5 years of age was estimated to be €4.82 billion, with 55% of global costs accounting for hospitalizations and 45% for outpatient costs (2017 estimates) (6).

RSV presents seasonally, with outbreaks of infection in the Northern Hemisphere usually lasting five months and occurring between October and March, while the peak of infections may vary between December and February (1, 4, 5).

The nature of RSV disease is unpredictable, and it is challenging to identify in advance which child will develop severe disease (7). Major risk factors for severe RSV disease and need for hospitalization are age <1 year and RSV strain virulence, which regards all children; additional risk factors are prematurity, congenital heart disease, chronic lung disease, Down Syndrome, neuromuscular impairment, and immunodeficiency (1, 8–14).

RSV-bronchiolitis lacks etiological prevention and treatment effective for all children. Clinical management is focused on supportive care, while pharmacological treatment with medicines such as bronchodilators, corticosteroids, and antibiotics have limited use in reducing disease severity and are currently not recommended by national guidelines (15–22). However, unnecessary medicines and non-evidence-based treatment are still common (23–27).

Several environmental and behaviour measures are effective in the prevention of RSV-bronchiolitis. Among the most relevant: washing hands before touching the child; cleaning and disinfecting toys, utensils and other frequently touched surfaces; keeping a smoke-free home, car and any space near the child; keeping the child away from people with colds; wearing masks during the RSV season (1). In addition, pharmacological prophylaxis through monoclonal antibodies (palivizumab) may be available only for children with specific comorbidities (28, 29).

Although there is currently no licensed vaccine or monoclonal antibody to protect all children against RSV available in routine medical practice, there are several candidates in the final phases of clinical trials and the European Medicines Agency recently recommended and authorized nirsevimab to protect all children at their first RSV season (30, 31). Furthermore, as recommended by the World Health Organization (WHO) and by the European Centre for Disease Prevention and Control (ECDC), health policymakers and National Immunization Technical Advisory Groups (NITAGs) should consider both new vaccines and monoclonal antibody anti-RSV to be included within routine immunization calendar, to facilitate the access to protect all children (32–35). Therefore, understanding the burden of RSV at the outpatient and inpatient level is vital to understand the medical need for prevention and supporting the implementation of future prevention strategies to protect all children against RSV.

The present study aims to investigate the epidemiologic burden of bronchiolitis in Italy and to describe the seasonality using electronic paediatric primary care data.

## Methods

### Pedianet database

Pedianet is a national population database that contains anonymous patient-level data of more than 500,000 children since 2004, corresponding to around 4% of the annual paediatric population in Italy (23, 36–38). For this study, we included data from 152 family paediatricians (FPs) from several Italian regions including, Friuli-Venezia Giulia, Liguria, Lombardia, Piemonte, Veneto, (North Italy 54%), Lazio, Marche, Toscana, (Centre Italy 14%), and Abruzzo, Campania, Sardegna, and Sicilia (South with Islands 32%), who use the same software (JuniorBit®) in their professional practice and who contributed to the database from January 1st 2012, to December 31st 2019, when the data were extracted on March 15th 2020 (23, 36–38).

According to the Italian national healthcare system, each child is assigned to a FP, who is the primary referral for health-related matters. In Italy, there is a tax-funded public health care system with universal health coverage, and patients do not incur any additional direct costs related to primary care visits (39). Pedianet database captures several patient-level information, including the reason for accessing healthcare, health status, demographic data, diagnosis, clinical symptoms (free text or ICD-9 CM codes), drug (Anatomical-Therapeutical-Chemical codes), specialist appointments, diagnostic procedures, hospital, or emergency room (ER) admissions, growth parameters, and clinical outcome data. Data are anonymized with a monthly update to a centralized database based in *Società Servizi Pediatrici*, the legal owner of Pedianet, in Padova, Italy. Informed consent is required from children's parents to enter the data in the database and to have Pedianet data linked to other databases, such as the vaccine registry database or the hospitalization database, using unique patient identifiers. Data are manually validated for study-specific conditions, and the accuracy of diagnosis data was verified (23, 36–38).

Pedianet database can be linked to regional hospitalization and regional immunization databases. For this study, the Pedianet database was linked from 2017 onward with the hospitalization database of the Veneto Region in Italy. Veneto is a Region in the North-East part of Italy, with a population of about 5 million inhabitants (23, 36–38).

### Study design, population and case definition

The retrospective database analysis estimated the incidence rate of bronchiolitis and RSV-related episodes in all children aged 0–24 months who were registered with one of the FPs collaborating with the Pedianet network from January 1st, 2012, to December 31st, 2019. Age and seasonality were analyzed as the main risk factors, while the role of additional risk factors, such as prematurity, was also assessed.

Children with less than two visits for any reason during the study period were excluded from the analysis, except those born during 2019, to have a more precise denominator in calculating the incidence, like in previous studies (23, 36, 37).

Case definitions are reported in the Supplementary Material. Briefly, cases coded for acute bronchiolitis (ICD9-CM codes 466.1, 466.11 or 466.19) and a free text search for a descriptive diagnosis using a previously validated algorithm (23). RSV-bronchiolitis cases were identified from RSV-specific diagnosis codes (ICD9-CM 466.11) or a descriptive or coded diagnosis of acute bronchiolitis (ICD9-CM codes 466.1, 466.19) and RSV infection (ICD9-CM 079.6).

LRTI cases, such as pneumonia and bronchitis, were also identified from coded diagnosis and free text fields to reduce the bias due to diagnosis misclassification.

### Outcomes and statistical analysis

The primary outcome is the medically attended bronchiolitis episode, identified through Pedianet outpatient records for paediatric visits and hospital records for ER visits and admissions. To avoid duplicates, all visits occurring within 30 days of the initial diagnosis were considered follow-up visits.

Other outcomes include LRTI and comorbidities (details on definitions used are reported in Supplementary Table S1) and RSV-specific LRTI. Children born preterm before 37 weeks of gestational age were defined as premature.

Incidence rates (IR) of bronchiolitis were expressed as the number of episodes per 1,000 person-years with the appropriate 95% confidence intervals (CI) and were stratified by age group, sex, calendar month, the month of birth, depending on RSV seasonality as in-season cases (October-March) vs. out of season (April-September) and by the four seasons of the year (autumn [October-December], winter [January-March], spring [April-June], summer [July-September]). The Mann-Kendall trend test was used to analyze the trend over time of bronchiolitis incidence. RSV-bronchiolitis prevalence was estimated by dividing the number of RSV-bronchiolitis by the total number of bronchiolitis; RSV-LRTIs prevalence was estimated by dividing the number of RSV-LRTIs by the total number of LRTIs.

The role of prematurity in bronchiolitis' onset was investigated and expressed as odds ratio with the appropriate 95% CI.

All analyses were performed using the Statistical Analysis System software (version 9.4; SAS Institute, Cary, North Carolina, USA). Statistical significance was set at *p* < 0.05.

## Results

### Bronchiolitis incidence

**Figure 1:**
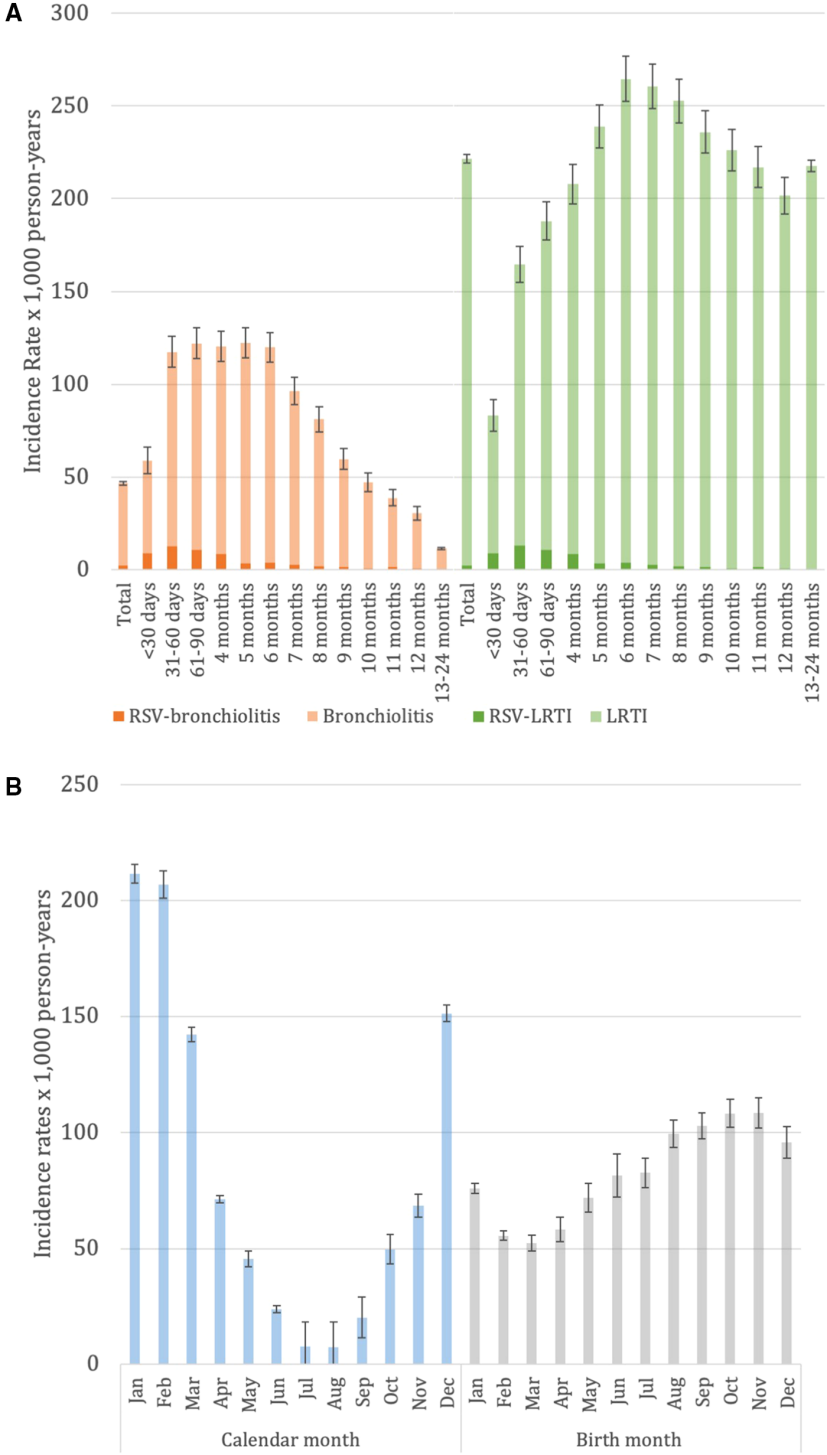
Panel **A**. Incidence rate of bronchiolitis (orange) and LRTI (green) stratified by RSV aetiology [RSV-coded-bronchiolitis (dark orange) and RSV-LRTI (dark green)] in children ≤24 months overall and stratified by age class. Panel **B**. Bronchiolitis IR in children aged ≤24 months by incident episode calendar month (light blue) and month of birth of the patient with bronchiolitis (grey). The whiskers represent 95% confidence intervals. Pedianet 2012–2019.

Out of 108,960 children aged ≤24 months included in the study cohort, 7,956 episodes of bronchiolitis were recorded among 6,955 (6.4%) children with at least one episode of bronchiolitis for an IR of 46.6 × 1,000 person-years (95% CI: 45.6–47.6). Of the total bronchiolitis episodes, 388 (5%) were defined as RSV-bronchiolitis for an IR of 2.3 × 1,000 person-years (95% CI: 2.1–2.5). However, the total number of bronchiolitis tested for pathogens is unknown (Figure 1 and Supplementary Table S2). The IR of bronchiolitis was the highest among males (55.7 vs. 39.9 episodes × 1,000 person-years in females) and in children aged between 1 month and six months (117.4 and 119.8 × 1,000 person-years, respectively), decreasing steadily in children aged ≥7 months. Similarly, the IR for RSV-bronchiolitis was the highest in children aged between 1 month and six months (12.7 and 3.8 × 1,000 person-years, respectively), decreasing steadily in children aged ≥7 months (Figure 1 and Supplementary Tables S2).

In children aged ≤24 months, the IR of bronchiolitis was higher during the winter season, January-March (IR 101.4 × 1,000 person-years) and lower during the summer season, July-September (IR 7.2 × 1,000); higher during the months in the RSV seasonality, October-March (IR 76.6 × 1,000 person-years) and lower during the months out of the RSV seasonality, April-September (IR 16.9 × 1,000 person-years) (Supplementary Table S2).

Considering the month of birth in children aged ≤24 months, IR slightly increased from March (52.3 × 1,000 person-years) to November (108.5 × 1,000 person-years); considering the calendar month, IR sharply increased from August (7.4 × 1,000 person-years) to January (IR 211.6 × 1,000 person-years) (Figure 1).

87% of bronchiolitis episodes (*N* = 6,961) were in children aged ≤12 months (IR: 83.9 × 1,000 person-years, 95% CI: 81.9–85.9). In this population, the IR of bronchiolitis was higher during the winter season, January-March (IR 186.3 × 1,000 person-years) and lower during the summer season, July-September (IR 11.8 × 1,000). IR bronchiolitis was higher during the months in the RSV seasonality, October-March (IR 137.77 × 1,000 person-years) and lower during the months out of the RSV seasonality, April-September (IR 29.55 × 1,000 person-years). The IRs in children aged ≤12 months were higher and almost doubled the relative IRs in children ≤24 months of age (IR_12 months_: 83.9 × 1,000 person-years, 95% CI: 81.9–85.9, vs. IR_24 months_: 46.6 × 1,000 person-years, 95% CI: 45.6–47.6) (Supplementary Figure S3).

### LRTI incidence

Out of 108,960 children aged ≤24 months included in the study cohort, 37,753 episodes of LRTIs (bronchiolitis, pneumonia and bronchitis) were recorded for an IR of 221.4 × 1,000 person-years (95% CI: 219.2 to 223.7); of these, 394 (1%) were caused by RSV. However, the total number of bronchiolitis tested for pathogens is unknown (Figure 1 and Supplementary Table S3). Similarly to bronchiolitis, also LRTI IR was higher among males than females (260.6 vs. 184.7 episodes × 1,000 person-years, respectively). LRTIs IR rapidly increased since the first months of life, with a peak at six months of life (273.0 × 1,000 person-years) and slowly decreased until 12 months of life (210.8 × 1,000 person-years), while was stable in the 13–24 months age range (220.3 × 1,000 person-years) (Figure 1 and Supplementary Table S3). The seasonality IRs and the calendar month IRs of LRTI had a pattern similar to bronchiolitis, with peaks between December and February, higher IRs in winter (387.87 × 1,000 person-years) and autumn (IR 260.12 × 1,000 person-years) while lower in summer (66.96 × 1,000 person-years), higher during the months in the RSV seasonality, October-March (IR 324.46 × 1,000 person-years) and lower during the months out of the RSV seasonality, April-September (IR 119.28 × 1,000 person-years) (Supplementary Figure S4 and Table S3). No marked variation between birth months was noted: LRTI IR ranged from a minimum of 304.78 (95% CI: 292.02 to 317.54) episodes×1,000 person-years in children born in December to a maximum of 346.69 (95% CI: 332.86 to 360.52) episodes×1,000 person-years in children born in June (Supplementary Figure S5).

49% of LRTI episodes (*N* = 18,487) were in children aged ≤12 months (IR: 221.9 × 1,000 person-years, 95% CI: 218.7–225.1). In this group, the IR of LRTIs was higher during the winter season, January-March (IR 416.33 × 1,000 person-years) and lower during the summer season, July-September (IR 57.55 × 1,000). LRTI IR was higher during the months in the RSV seasonality, October-March (IR 330.23 × 1,000 person-years) and lower during the months out of the RSV seasonality, April-September (IR 112.64 × 1,000 person-years). All-season and seasonality IRs in children aged ≤12 months were similar to the relative IRs in children ≤24 months of age (IR_12 months_: 221.9 × 1,000 person-years, 95%CI: 218.7–225.1, vs. IR_24 months_: 221.4 × 1,000 person-years, 95%CI: 219.2–223.7) (Supplementary Figure S6).

### Additional known risk factors: role of comorbidities and prematurity

97.5% of bronchiolitis episodes (7,760/7,956) happened in children with no comorbidities, while 196 episodes (2.5%) happened in children with comorbidities: the most frequent underlying clinical condition was a concomitant cardiovascular disease, present in 107 (1.3%) of the bronchiolitis episodes (Table 1).

**Table 1:** Clinical characteristics of patients ≤24 months with bronchiolitis. Pedianet 2012–2019.

	Bronchiolitis episodes	
	*N* (*N*_tot _= 7,956)	(%)
**Comorbidities**		
Cardiovascular	107	(1.34)
Neuromuscular—nervous	47	(0.59)
Pulmonary	12	(0.15)
Immunosuppressive disorders or therapies	8	(0.10)
Others	22	(0.27)
**Total episodes in children with comorbidities**	196	(2.46)
**Total episodes in children with no comorbidities**	7,760	(97.54)
**Prematurity (i.e., <37 gestational weeks)**		
33≤ gestational weeks <37	486	(6.11)
28≤ gestational weeks ≤32	111	(1.40)
≤27 gestational weeks	27	(0.34)
**Total episodes in children born prematurely**	624	(7.84)
**Total episodes in children born at term**	7,332	(92.16)

Regarding gestational age, 92.2% of bronchiolitis episodes (7,332/7,956) happened in children born at term, while 624 episodes (7.8%) happened in children born at <37 gestational weeks: in 486 cases (6.1%) children were born moderate to late preterm, having a gestational age between 33 and 37 weeks, while in 138 cases (1.7%) children were born with ≤32 gestational weeks.

Being born preterm increased the odds of having bronchiolitis: children with a gestational age between 33 and 37 weeks showed an OR 1.41 (95% CI: 1.27–1.57); children with a gestational age between 28 and 32 weeks showed an OR 1.90 (95% CI: 1.52–2.37); while children with a gestational age ≤27 weeks showed an OR 1.89 (95% CI: 1.20–2.98).

## Discussion

This study assessed the burden of bronchiolitis and LRTI in Italy over eight consecutive years using a large paediatric database. Our results show that bronchiolitis and LRTI are responsible for a significant burden in children aged ≤24 months, while bronchiolitis IR are twofold higher in children aged ≤12 months. IRs of both bronchiolitis and LRTI were higher in the winter season and lowered in the summer season; higher in RSV seasonality, October-March, and lower out the RSV seasonality, April-September. None to little variation was seen in the IRs of bronchiolitis and LRTI by the month of birth.

Our results are coherent with other epidemiological studies, recently conducted both in Italy and in the Northern hemisphere, that showed that most children with RSV infection requiring a medical visit at a paediatric practice or hospitalization were born at term and healthy (8–11): this confirms that RSV disease is unpredictable and that is very difficult to identify in advance which child will develop severe disease (7–11). Furthermore, although prematurity increased the odds of having bronchiolitis, in particular in those born severe to extreme preterm (≤32 gestational weeks), our results confirmed that the majority of bronchiolitis cases (92.2%) happened in children born at term. Furthermore, although children with comorbidities showed an increased risk of bronchiolitis, our results confirmed that most bronchiolitis cases (97.5%) happen in children with no comorbidities or otherwise healthy.

Regarding RSV infection, only 388 of the 7,956 bronchiolitis episodes were coded as RSV-related (5%). However, the total number of bronchiolitis tested for pathogens is unknown. RSV is well known to be the most common infecting agent in bronchiolitis among children ≤5 years old, and it accounts for 50% to 80% of hospitalization for bronchiolitis in children aged 0–5, and it rises to 80% in children aged ≤12 months (2). Furthermore, 40% of hospitalization for pneumonia in children aged ≤12 months are also due to RSV (3). Nevertheless, laboratory testing is predominantly conducted at the hospital level (either during an emergency room visit or in case of hospitalization), while at the outpatient level, laboratory testing is rare (40).

A gestational age ≤35 weeks is considered an eligibility criterion for RSV bronchiolitis prevention with the specific anti-RSV monoclonal antibody palivizumab in the Italian and Spanish guidelines, although many Regions of both countries have restricted the eligibility to 32 and 29 gestational weeks (20, 41, 42), while the American guidelines recommend a threshold of ≤29 weeks of gestational age (21).

Our study confirms that all children in their first RSV season are at risk of bronchiolitis and LRTI, regardless of the month of birth, and would support strategies to protect all children against RSV disease. Although, to date, there is no available vaccine or monoclonal antibody in the routine medical practice to protect all children against RSV, several preventive products are in the final stages of clinical trials, while a new long-acting anti-RSV monoclonal antibody, nirsevimab, was recently recommended and authorized by EMA to protect all children at their first RSV season (30, 31). Therefore, health policymakers should consider including these new preventive options, once available, within the routine immunization calendar to facilitate access to protect all children, as already recommended by WHO and ECDC (32–35).

To our knowledge, this is the first study assessing the burden of bronchiolitis and LRTI with insights into the RSV seasonality at the outpatient level in Italy. The strengths of our study are its size, generalizability and representative coverage of paediatric patients in Italy.

There are limitations in estimating RSV incidence due to the poor RSV testing available at the outpatient level. Currently, there is no systematic national surveillance of RSV available in Italy, although a project to include it within the National influenza-like illness Surveillance System (INFLUNET) is ongoing (43) and a project to monitor RSV season using the more precise definition of acute LRTI is also about to start (44). Most RSV-bronchiolitis and RSV-LRTI at the outpatient level are not identified, leading to an underestimation of the actual paediatric burden of RSV in Italy. In fact, previous international studies conducted in the Northern Hemisphere reported outpatient IRs of RSV-bronchiolitis and RSV-LRTIs > 200 × 1,000 person-years (4, 5), while in our study was only 2.27 and 2.31 × 1,000 person-years, respectively. Also, FPs might not report the aetiology of bronchiolitis and LRTI in the database, knowing that RSV is the leading viral cause of bronchiolitis and LRTI in children ≤24 months, further diminishing the number of RSV infections detected.

## Conclusions and future implications

In conclusion, our study supports the evidence that bronchiolitis and LRTI waves in Italy usually last five months and present seasonally, between October and March, with a peak between December and February. The major risk factors of bronchiolitis and LRTI are age ≤12 months and age ≤24 months, respectively, and seasonality, with all children being at risk at their first RSV season within October-March, regardless of the month of birth. Although children born preterm and those with comorbidities had a higher risk of bronchiolitis, most cases happened in those born at term (92.2%) and in children with no comorbidities or otherwise healthy (97.5%). Information on RSV-bronchiolitis and RSV-LRTI in the outpatient setting is still lacking, and the actual burden is not fully recognized and may be underestimated by health policy makers^2^. Strengthening RSV surveillance at the outpatient and inpatient level may unveil the actual burden of RSV disease in children, supporting policymakers in implementing appropriate preventive strategies to protect all children against RSV.

Interventions aimed at preventing RSV disease, including introducing anti-RSV vaccines and long-acting monoclonal antibodies, would impact the incidence of bronchiolitis and LRTI in our setting. In order to facilitate universal access to protecting all children from RSV, WHO and ECDC recommended that the National Immunization Advisory Group (NITAGs) consider including these preventive tools within the routine immunization calendar. Further studies focused on the burden of RSV at outpatient and inpatient levels are needed to unveil the true burden of this disease and its impact in the post-Covid19 era. Indeed, having a surveillance system of respiratory pathogens, including RSV, will support health policymakers in implementing and evaluating the effectiveness of those new preventive strategies when they are finally available to protect all children against RSV.

## Data Availability

The data used in this study cannot be made publicly available due to Italian data protection laws. The anonymized datasets generated during and/or analyzed during the current study can be provided on reasonable request, from the corresponding author, after written approval by the Internal Scientific Committee (info@pedianet.it).
