# Supplementary material for: Assessing the burden of bronchiolitis and lower respiratory tract infections in children ≤24 months of age in Italy, 2012–2019
Source: Front Pediatr. 2023 May 5;11:1143735. doi: 10.3389/fped.2023.1143735 (PMC10196108; doi:10.3389/fped.2023.1143735)

Supplementary material

Episode definition:

Bronchiolitis episode is defined as first visit with a bronchiolitis diagnosis identified with

- an International Classification of Diseases, 9th Revision, Clinical Modification code (ICD-9-CM):
  - acute bronchiolitis (ICD-9-CM code 466.1, 466.11 or 466.19)

OR

- a descriptive diagnosis, retrieved searching free text fields:
  - description of first episode of respiratory distress with at least two of the following symptoms coryza, cough, wheezing or crackles, and tachypnea or chest retractions, skin color changes, nasal flaring or fever [8,9]

RSV-bronchiolitis is defined as first visit with a bronchiolitis diagnosis identified with

- an International Classification of Diseases, 9th Revision, Clinical Modification code (ICD-9-CM):
  - acute bronchiolitis (ICD-9-CM code 466.1, 466.11 or 466.19)

    AND
  - RSV infection (ICD-9-CM code: 079.6, Respiratory syncytial virus (RSV))

OR

- a descriptive diagnosis, retrieved searching free text fields:
  - description of first episode of respiratory distress with at least two of the following symptoms coryza, cough, wheezing or crackles, and tachypnea or chest retractions, skin color changes, nasal flaring or fever [8,9]

    AND
  - RSV infection (ICD-9-CM code: 079.6, Respiratory syncytial virus (RSV))

Non-specific bronchiolitis is defined as a first visit with a bronchiolitis diagnosis identified with

- an International Classification of Diseases, 9th Revision, Clinical Modification code (ICD-9-CM):
  - acute bronchiolitis (ICD-9-CM code 466.1, 466.11 or 466.19)

OR

- a descriptive diagnosis, retrieved searching free text fields:
  - description of first episode of respiratory distress with at least two of the following symptoms coryza, cough, wheezing or crackles, and tachypnea or chest retractions, skin color changes, nasal flaring or fever [8,9]

WITHOUT a mention of

- RSV infection (ICD-9-CM code: 079.6, Respiratory syncytial virus (RSV))

Pneumonia diagnosis is defined as first visit with pneumonia identified with

- an International Classification of Diseases, 9th Revision, Clinical Modification code (ICD-9-CM):
  - pneumonia (ICD-9-CM code 485, 486)

OR

- a descriptive diagnosis, retrieved searching free text fields:
  - description of lower respiratory tract infection with the presence of i) fever at the moment of visit (core body temperature > 38°C), ii)

    AND
  - at least one symptom (cough, sputum production, pleuritic pain, poor feeding), iii)

    AND
  - at least one sign (tachypnea, dyspnea, wheezing, inspiratory crackles, bronchial breathing, pleural rub) for less than 14 days, iv)

    AND/OR
  - new or increasing alveolar infiltrate in chest radiography (if available).

RSV-Pneumonia diagnosis is defined as first visit with pneumonia identified with

- an International Classification of Diseases, 9th Revision, Clinical Modification code (ICD-9-CM):
  - Pneumonia due to RSV (ICD-9-CM code 480.1)

OR

  - pneumonia (ICD-9-CM code 485, 486)

    AND
  - RSV infection (ICD-9-CM code: 079.6)

OR

- a descriptive diagnosis, retrieved searching free text fields:
  - description of lower respiratory tract infection with the presence of i) fever at the moment of visit (core body temperature > 38°C), ii)

    AND
  - at least one symptom (cough, sputum production, pleuritic pain, poor feeding), iii)

    AND
  - at least one sign (tachypnea, dyspnea, wheezing, inspiratory crackles, bronchial breathing, pleural rub) for less than 14 days, iv)

    AND/OR
  - new or increasing alveolar infiltrate in chest radiography (if available)

    AND
  - RSV infection (ICD-9-CM code: 079.6)

Bronchitis diagnosis is defined as first visit with bronchitis identified with

- an International Classification of Diseases, 9th Revision, Clinical Modification code (ICD-9-CM):
  - acute bronchitis (ICD-9-CM code 466.0)

<u>RSV-Bronchitis</u> diagnosis is defined as first visit with bronchitis identified with

- an International Classification of Diseases, 9th Revision, Clinical Modification code (ICD-9-CM):
    - acute bronchitis (ICD-9-CM code 466.0)

      AND
    - RSV infection (ICD-9-CM code: 079.6, Respiratory syncytial virus (RSV))

Table 1s. Comorbidities and risk factors considered. Pedianet 2012-2019

Risk Factors - Diagnostic Group	Code
Pulmonary	
Cystic fibrosis	277.0
Bronchopulmonary dysplasia	770.7
Bronchiectasis	494
Chronic bronchitis	491
Chronic respiratory disease or failure	518.83, 518.84, 519.9
Congenital anomalies of respiratory system	748
Nervous - Neuromuscular	
Cerebral degenerations usually manifest in childhood	330
Anterior horn cell disease	335
Infantile cerebral palsy	343
Hereditary and idiopathic peripheral neuropathy	356
Myoneural disorders	358
Muscular dystrophies and other myopathies	359.0-359.23, 359.9, 775.2
Spina bifida and other congenital anomalies of nervous system<br>Psychomotor retardation	740 – 742, 308.2
Cardiovascular	
Congenital heart disease	745.0 – 747.4, 747.83
Heart failure	428
Chronic pulmonary heart disease	416
Cardiomyopathy	425.1-425.4, 429.1, 429.3
Immunosuppressive disorders or therapies	
HIV infection	042 (V08)
Malignancy	140-208
Hereditary immunodeficiency	279
Organ or bone marrow transplantation	V42
Radiation or chemotheraperson-years	V58.0-V58.1
Asplenia	759.0
Diseases of white blood cells	288
Others	
Down’s syndrome	758.0
Other chromosomal anomalies	758.1-758.9
Congenital infections	090, 771.0-771.2
Congenital deformities of the spine	754.2, 756.1
Metabolic disorders	270 – 272, 277

Table 2s. Incidence rates of bronchiolitis and RSV-bronchiolitis in children aged 0-2 years overall and stratified by sex, age class, season and in-season vs. out-season. Pedianet 2012-2019

	No. of bronchiolitis episodes (N=7,956)	Bronchiolitis Incidence Rate x 1,000 person-years (95% IC)	No. of RSV-bronchiolitis episodes (N=388)	RSV-bronchiolitis Incidence Rate x 1,000 person-years (95% IC)
Total	7,956	46.57 (45.55 - 47.6)	388	2.27 (2.05 - 2.5)
Sex				
Male	4,611	55.74 (54.13 - 57.34)	208	2.51 (2.17 - 2.86)
Female	3,345	37.97 (36.68 - 39.26)	180	2.04 (1.74 - 2.34)
Age class				
<30 days	260	58.89 (51.74 - 66.05)	39	8.83 (6.06 - 11.61)
31-60 days	765	117.41 (109.09 - 125.73)	83	12.74 (10.00 - 15.48)
61-90 days	835	122.03 (113.75 - 130.31)	73	10.67 (8.22 - 13.12)
4 months	835	120.23 (112.07 - 128.38)	59	8.5 (6.33 - 10.66)
5 months	857	122.21 (114.03 - 130.39)	24	3.42 (2.05 - 4.79)
6 months	846	119.8 (111.73 - 127.87)	27	3.82 (2.38 - 5.27)
7 months	684	96.4 (89.18 - 103.63)	19	2.68 (1.47 - 3.88)
8 months	578	81.08 (74.47 - 87.69)	14	1.96 (0.94 - 2.99)
9 months	427	59.62 (53.97 - 65.28)	10	1.4 (0.53 - 2.26)
10 months	338	47 (41.99 - 52.01)	6	0.83 (0.17 - 1.5)
11 months	280	38.76 (34.22 - 43.3)	10	1.38 (0.53 - 2.24)
12 months	257	30.41 (26.69 - 34.12)	5	0.59 (0.07 - 1.11)
13-24 months	994	11.32 (10.62 - 12.03)	19	0.22 (0.12 - 0.31)
Season				
Autumn [1Oct-31Dec]	2,172	51.36 (49.2 - 53.52)	91	2.15 (1.71 - 2.59)
Winter [1Jan-31Mar]	4,338	101.4 (98.38 - 104.42)	254	5.94 (5.21 - 6.67)
Spring [1Apr-30Jun]	1,137	26.44 (24.91 - 27.98)	39	0.91 (0.62 - 1.19)
Summer [1Jul-30Sep]	309	7.23 (6.42 - 8.03)	4	0.09 (0 - 0.19)
In season vs out of season				
In season [October 1st - 31 Mar]	6,510	76.54 (74.69 - 78.4)	345	4.06 (3.63 - 4.48)
Out of season [April 1st-September 30th]	1,446	16.86 (15.99 - 17.73)	43	0.5 (0.35 - 0.65)

Figure 2s. Bronchiolitis IR by calendar month in children aged 0-2 years. Pedianet 2012-2019

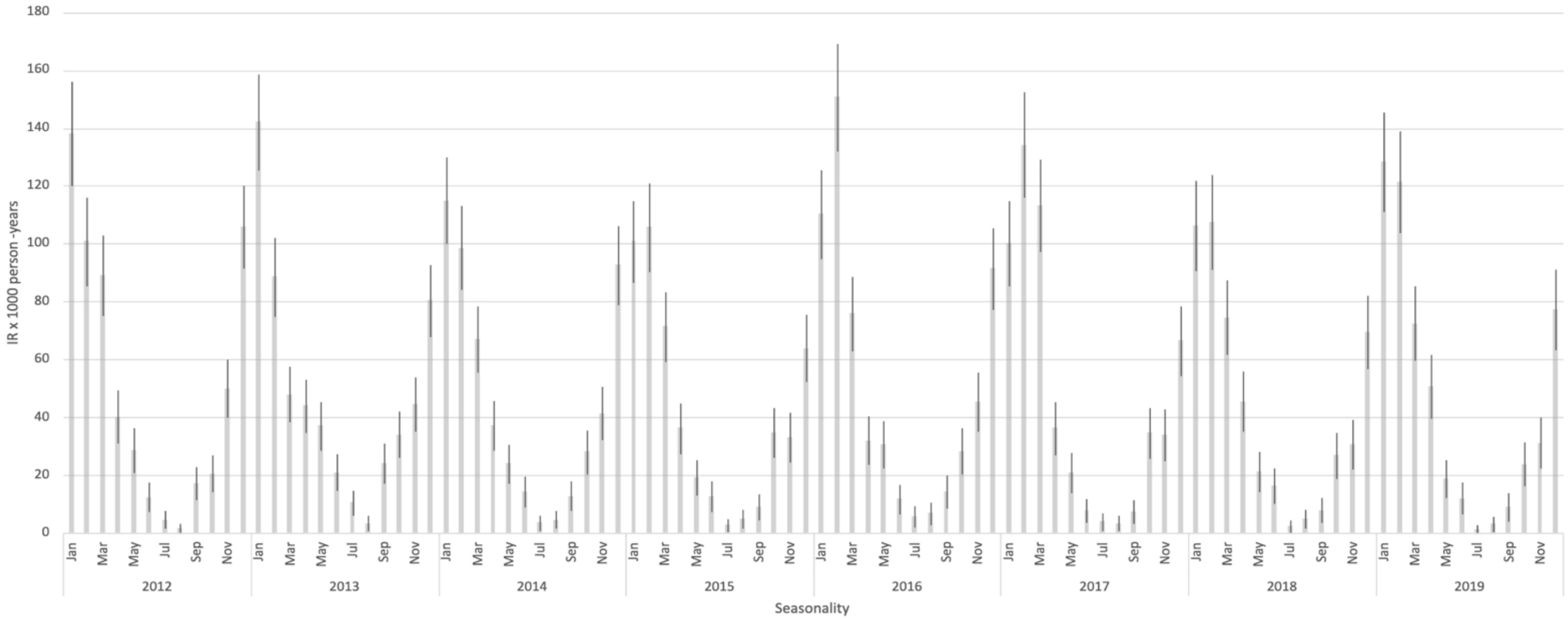

Figure 3s. Bronchiolitis IR in children aged < 1 year, overall and stratified by season, in-season vs. out-season, calendar month of the episode and birth month of the child. Pedianet 2012-2019

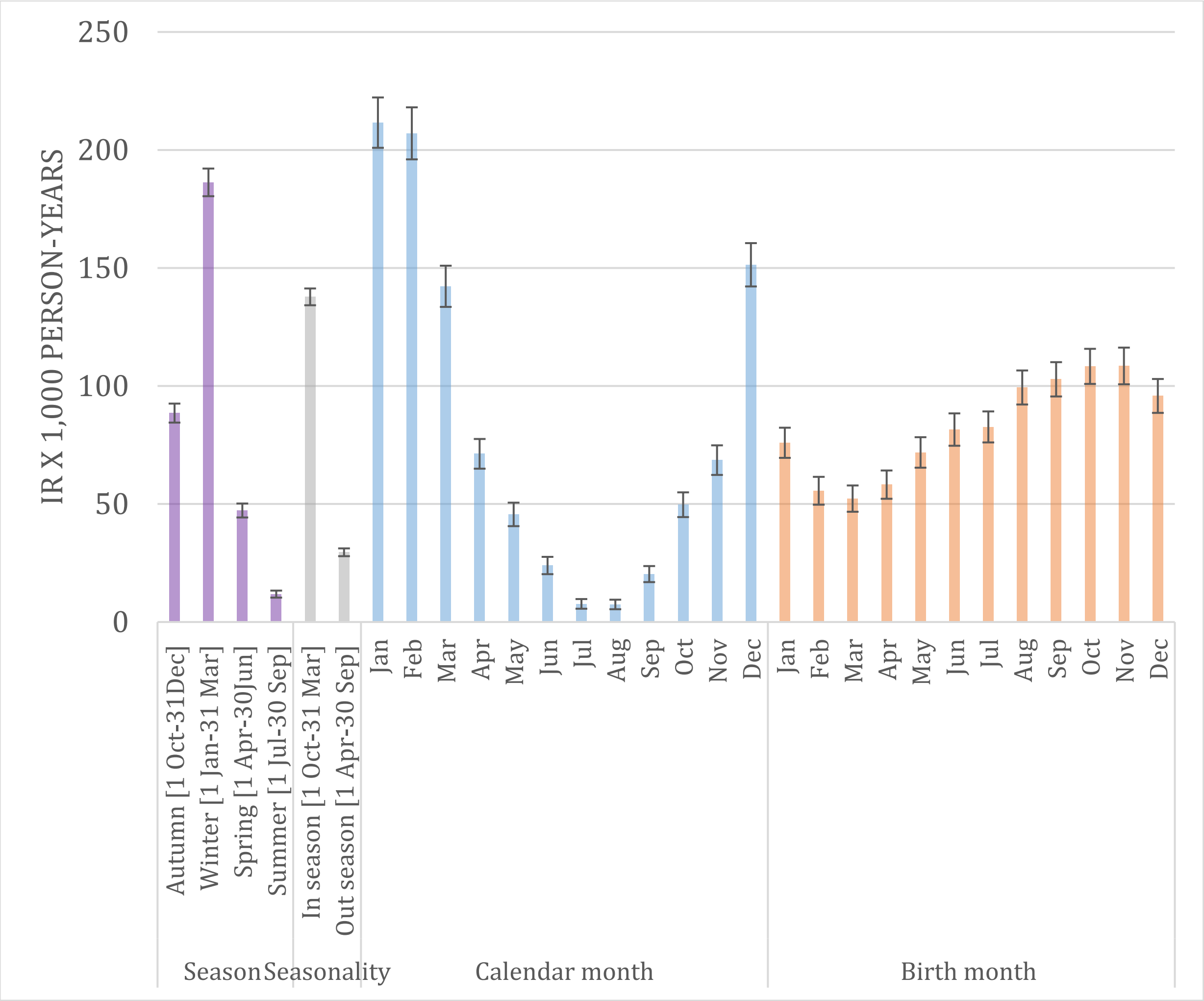

Table 3s. Incidence rates of LRTI and RSV-LRTI in children aged 0-2 years overall and stratified by sex, age class, season and in-season vs. out-season. Pedianet 2012-2019

	No. of LRTI episodes (N=37,827)	LRTI incidence rate x 1,000 person-years (95% IC)	No. of RSV-LRTI episodes (N=394)	RSV-LRTI incidence rate x 1,000 person-years (95% IC)
Total	37,827	221.44 (219.21 - 223.67)	394	2.31 (2.08 - 2.53)
Sex				
Male	21,558	260.59 (257.11 - 264.06)	213	2.57 (2.23 - 2.92)
Female	16,269	184.67 (181.84 - 187.51)	181	2.05 (1.76 - 2.35)
Age class				
<30 days	366	82.91 (74.41 - 91.4)	39	8.83 (6.06 - 11.61)
31-60 days	1,050	161.15 (151.4 - 170.89)	85	13.05 (10.27 - 15.82)
61-90 days	1,281	187.21 (176.96 - 197.46)	75	10.96 (8.48 - 13.44)
4 months	1,459	210.07 (199.29 - 220.85)	59	8.5 (6.33 - 10.66)
5 months	1,733	247.13 (235.5 - 258.77)	24	3.42 (2.05 - 4.79)
6 months	1,928	273.02 (260.83 - 285.21)	27	3.82 (2.38 - 5.27)
7 months	1,916	270.05 (257.95 - 282.14)	19	2.68 (1.47 - 3.88)
8 months	1,872	262.61 (250.71 - 274.51)	14	1.96 (0.94 - 2.99)
9 months	1,762	246.03 (234.55 - 257.52)	10	1.4 (0.53 - 2.26)
10 months	1,701	236.52 (225.28 - 247.76)	6	0.83 (0.17 - 1.5)
11 months	1,637	226.6 (215.62 - 237.58)	10	1.38 (0.53 - 2.24)
12 months	1,782	210.83 (201.04 - 220.62)	5	0.59 (0.07 - 1.11)
13-24 months	19,340	220.33 (217.22 - 223.43)	21	0.24 (0.14 - 0.34)
Season				
Autumn [1Oct-31Dec]	11,001	260.12 (255.26 - 264.98)	93	2.2 (1.75 - 2.65)
Winter [1Jan-31Mar]	16,594	387.87 (381.97 - 393.78)	257	6.01 (5.27 - 6.74)
Spring [1Apr-30Jun]	7,369	171.38 (167.47 - 175.3)	40	0.93 (0.64 - 1.22)
Summer [1Jul-30Sep]	2,863	66.96 (64.51 - 69.41)	4	0.09 (0 - 0.19)
In season vs out season				
In season [1 Oct-31 Mar]	27,595	324.46 (320.64 - 328.29)	350	4.12 (3.68 - 4.55)
Out season [1 Apr-30 Sep]	10,232	119.28 (116.97 - 121.59)	44	0.51 (0.36 - 0.66)

Figure 4s. LRTI IR by calendar month in children aged 0-2 years. Pedianet 2012-2019

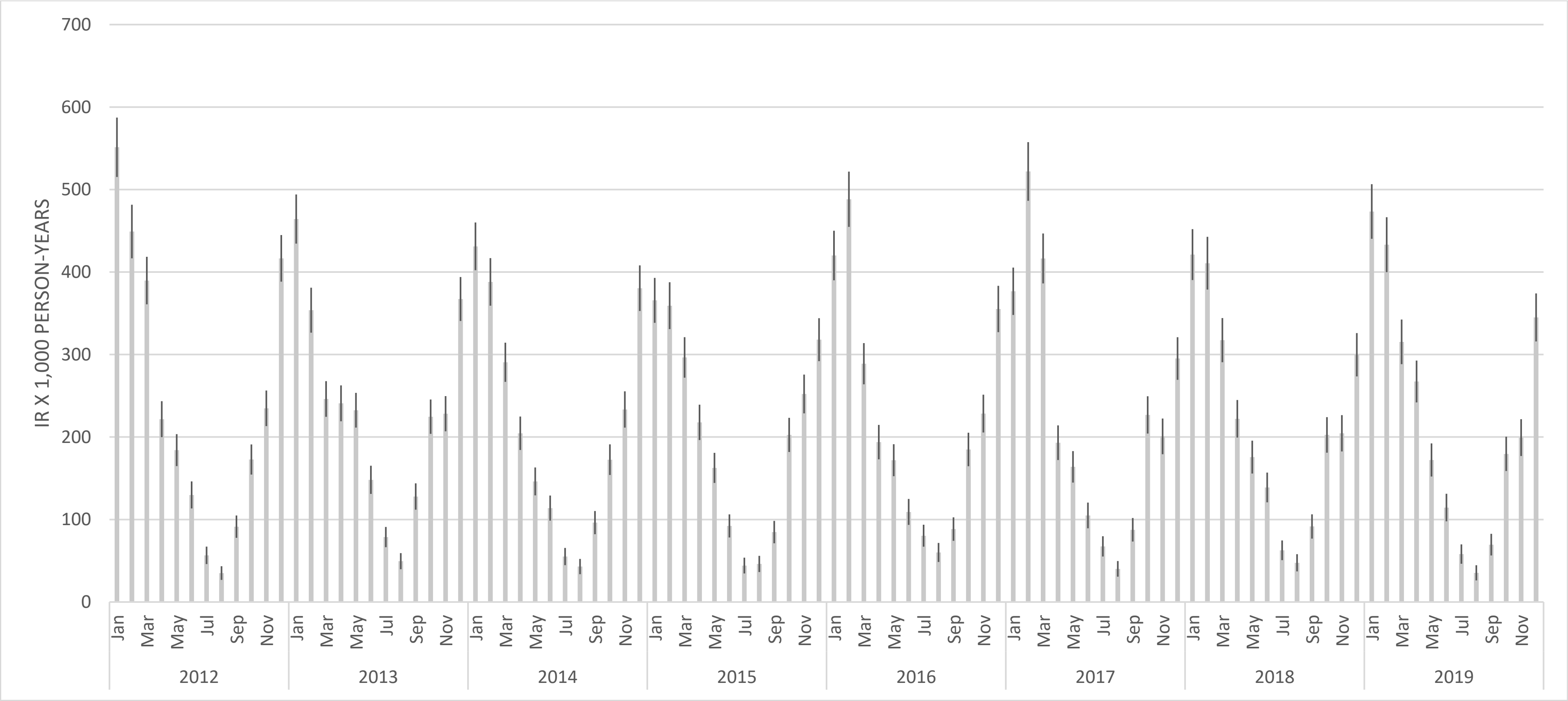

Figure 5s. LRTI IR in children aged 0-2 years by month of birth of the patients with LRTI and classified as in-season and out-season. Pedianet 2012-2019

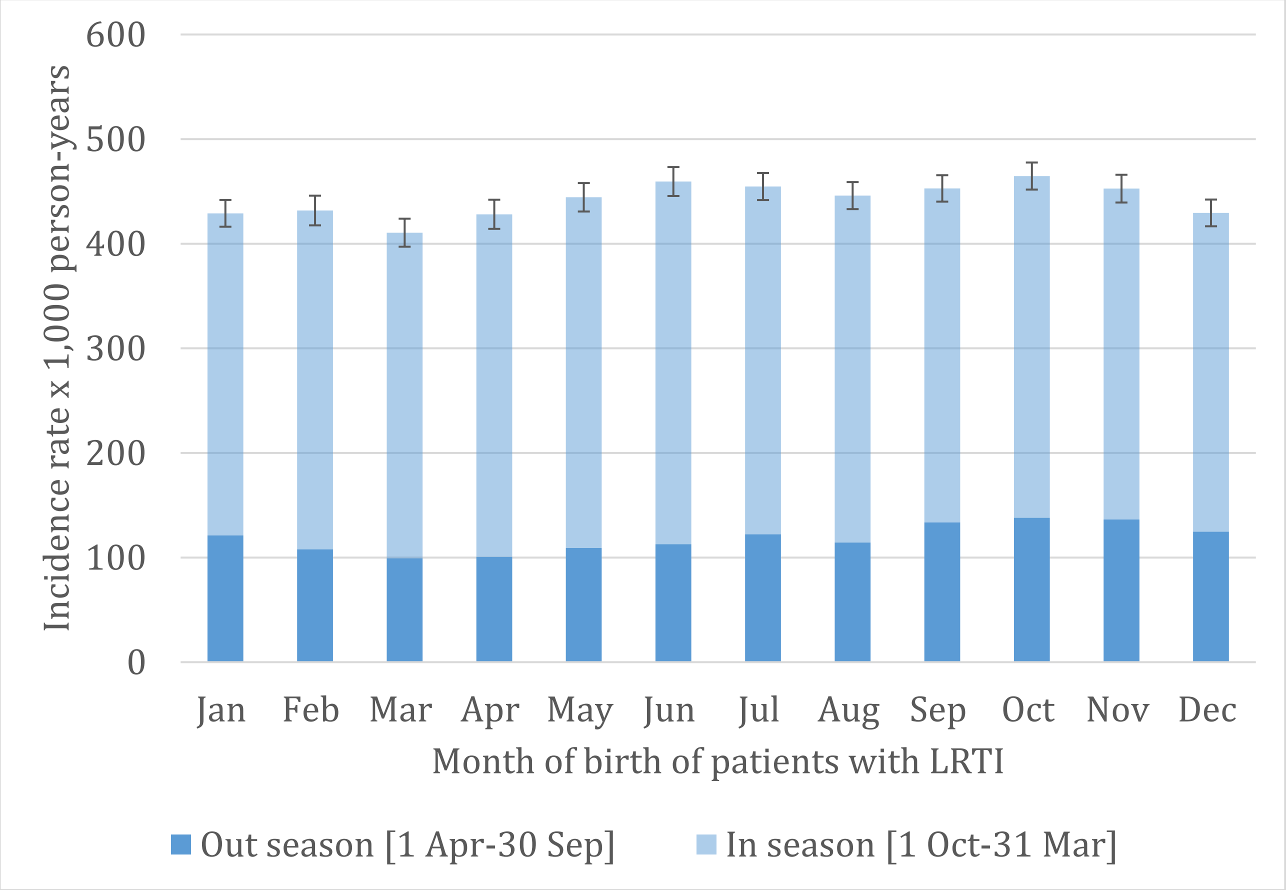

Figure 6s. LRTI IR in children aged < 12 months overall stratified by season, in-season vs. out-season, calendar month and patient birth month. Pedianet 2012-2019

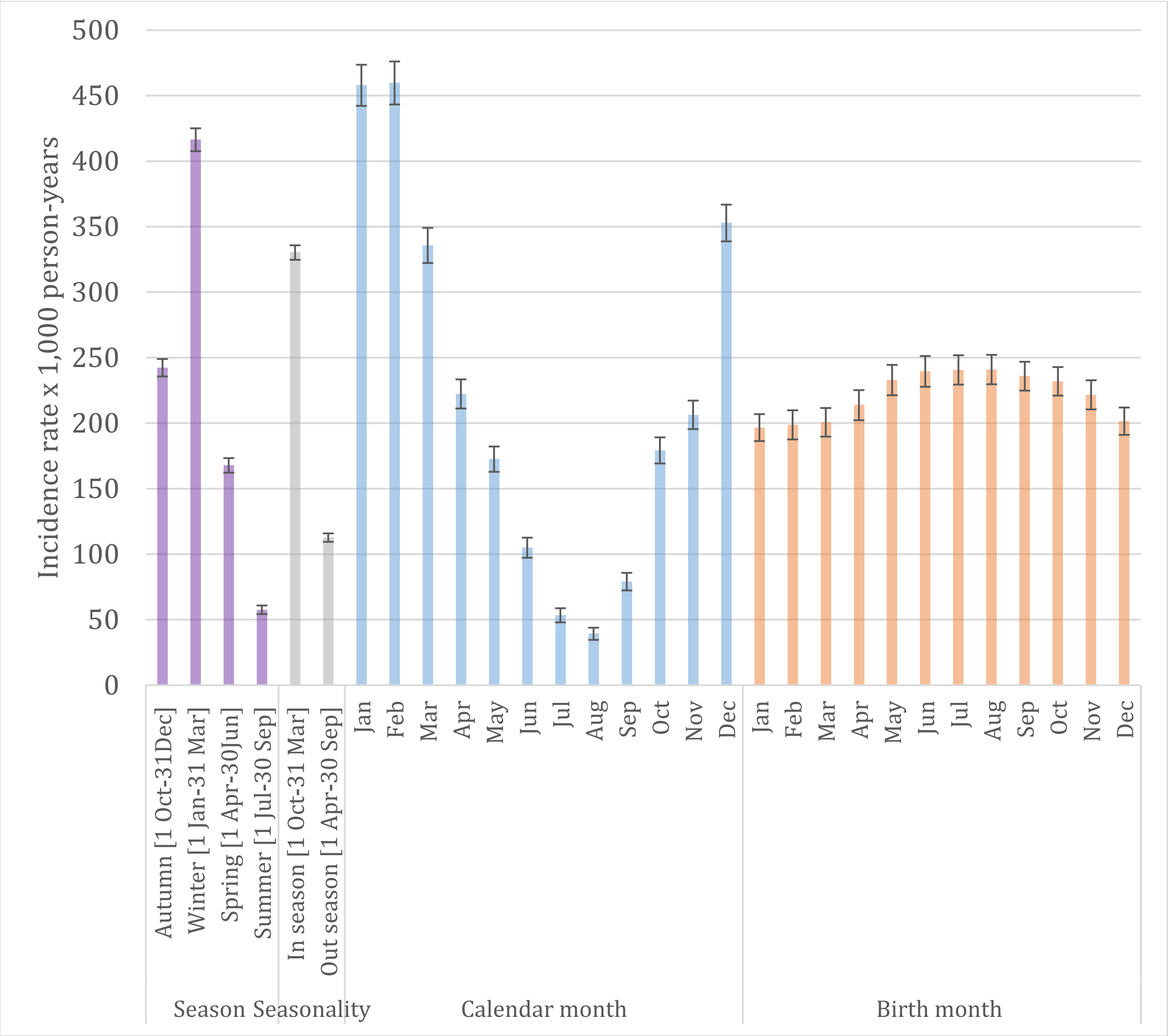

Supplement: Supplementary file 1 [file Datasheet1.pdf]
